# Genetic Association With *Pseudomonas aeruginosa* Acquisition in Cystic Fibrosis: Influence of Surfactant Protein D and Mannose-Binding Lectin

**DOI:** 10.3389/fimmu.2021.587313

**Published:** 2021-02-19

**Authors:** Nasenien Nourkami-Tutdibi, Klemens Freitag, Michael Zemlin, Erol Tutdibi

**Affiliations:** Saarland University Medical Center, Hospital for General Pediatrics and Neonatology, Homburg, Germany

**Keywords:** cystic fibrosis, *Pseudomonas aeruginosa*, surfactant protein D, SFTPD, mannose-binding protein, MBL, biomarker, gene polymorphisms

## Abstract

**Background:** *Pseudomonas aeruginosa* (PA) infection in cystic fibrosis (CF) is associated with poor prognosis. Surfactant protein-D (SFTPD) and mannose-binding lectin (MBL) play a critical role in innate immunity and response to bacterial infections. We investigated serum levels and genetic variants of SFTPD and MBL in CF patients.

**Method:** Thirty-five Caucasian patients homozygous for ΔF508del were genotyped for functional relevant polymorphisms within MBL2 (promoter−221 Y/X, codons 52, 54, and 57) and SFTPD genes (Met11Thr, Ala160Thr, and Ser270Thr). Serum levels of collectins, clinical characteristics, and PA status were correlated with genetic data.

**Results:** Patients age, gender, and PA status did not affect MBL and SFTPD serum concentrations. MBL concentrations were correlated with MBL haplotypes. Patients with chronic *Pseudomonas aeroginosa* infection (PAC) and MBL insufficiency had a shorter interval between first PA infection and onset of PAC (0.01 vs. 4.6 years, *p* < 0.04) as well as a lower median age at transition to PAC (9.8 vs. 16.4 years, *p* < 0.03) compared to MBL sufficient patients with PAC. SFTPD serum level and FEV1% (Spearman *r* = −0.41, *p* < 0.03) showed a negative correlation irrespective of PA infection status. The hazard ratio to PA acquisition was increased in carriers of the SFTPD haplotype 11Thr-160Ala-270Ser compared to carriers of the common 11Met-160Thr-270Ser haplotype [HR 3.0 (95%CI: 1.1–8.6), *p* < 0.04].

**Conclusion:** MBL insufficiency leads to a shorter interval between first PA infection and onset of chronic infection. Susceptibility to PA acquisition is associated with SFTPD genetic variants with 11Thr-160Ala-270Ser as risk haplotype for early PA infection. This may be due to presence of threonine associated with oligomeric structure of SFTPD and binding ability to bacteria.

## Introduction

Patients with cystic fibrosis (CF) are particularly prone to develop recurring pulmonary infections with a damaging local inflammatory response, leading to progressive respiratory insufficiency and organ failure. Impaired mucociliary clearance resulting in chronic bacterial airway infection is a hallmark of CF and chronic infection is the main cause of pulmonary damage and premature death (1). *Pseudomonas aeruginosa* (PA) is the most prevalent microorganism contributing to persistent airway infection and chronic inflammation in the pathogenesis of CF lung disease (2). Progressive pulmonary disease associated with chronic PA airway infection and inflammation is the major cause of morbidity and mortality in CF patients (3). Early acquisition of this pathogen is a strong predictor for a worse prognosis with poorer survival (4). There is a considerable variation in the phenotype and course of the disease in CF even in patients with the same cystic fibrosis transmembrane conductance regulator (CFTR) genotype, suggesting that other factors are important for their prognosis. There are more than 2,000 known variants in the CFTR gene with ΔF508del being most common variant causing for two third of all CF diseases (5).

The susceptibility to PA infection and chronic colonization is modulated by various factors as genetic modifiers have an impact on predisposition to infections with PA in CF patients (6). Several studies indicate that viral co-infections do have an important impact on exacerbation and lung deterioration and that immune response to viral infections is modulated in CF patients colonized with PA (7, 8).

Pulmonary collectins such as surfactant protein-D (SFTPD) and mannose-binding lectin (MBL) have been identified as key factors in the innate host immunity and regulation of immune reaction to pathogens in the lung (9). MBL is a first line host defense protein of importance for the innate immunity (10, 11). Polymorphisms in the MBL gene named MBL2 may result in unstable and low functional levels of MBL proteins. MBL deficiency and MBL2 gene polymorphisms are associated with an opsonization defect and are linked to an increased risk of lung infection and to deteriorating lung function in patients with CF (12). Patients with CF and genetic MBL insufficiency show reduced lung function and earlier infection with PA (13).

Several surfactant proteins are also involved in surfactant function and innate immunity in the human lung (14–16). Both lung function and innate immunity are altered in CF (17, 18). Changes in SFTPD secretion or degradation may potentially influence the course of CF lung disease (19). SFTPD is a soluble collagenous C-type lectin with important anti-microbial and anti-inflammatory properties. Although it is subject to functionally relevant modification by common polymorphisms and unregulated inflammation, the functional status of SFTPD in CF remains unclear and studies about single-nucleotide polymorphisms (SNPs) of SFTPD coding gene in CF are very sparse (18, 20). A number of studies analyzed SFTPD levels in serum of CF patients. They demonstrated that serum SFTPD levels in CF patients are significantly elevated in comparision to healthy controls (18, 21). A positive correlation with lung inflammation and a negative with impaired lung function could be demonstrated (9). Since then, SFTPD serum levels have been discussed as a biomarker for inflammation and disease progression in patients with chronic lung disease (22). Studies analyzing genetic polymorphisms for SFTPD gene in CF patient are still sparse though.

The aim of this study was to determine whether functionally relevant MBL2 and SFTPD polymorphisms are associated with age at detection of first PA infection and onset of PA colonization in CF patients. In addition, we aimed to analyze whether differences in SFTPD and MBL2 haplotypes may explain individual variabilities of CF patients in susceptibility to PA infection.

## Methods

### Study Population and Data Collection

In this retrospective study, we enrolled children with CF who were followed between 1988 and 2018 at our Cystic Fibrosis Center, Homburg (Germany). The diagnosis of CF based on typical clinical features, abnormal sweat chloride test and the identification of mutations in the CFTR gene. As a part of the clinical follow-up and to confirm the CF diagnosis, all patients were routinely tested for the most common CTFR mutations using allele-specific oligonucleotide hybridization methods (CF StripAssay EXT, ViennaLab Diagnostics, Vienna, Austria) at the local human genetic diagnostic laboratory. The patients were seen regularly in the center at least four times a year. Sputum and deep throat swab samples were cultured at each visit in order to detect dysbiosis. Pulmonary function tests were performed in children above 6–8 years of age. All participants or in case of minors their parents provided written informed consent in accordance with regulations of the local institutional review boards (file no 220/05). We included only those CF patients into the study, if (1) patient was homozygous for the ΔF508del allele, (2) stored sample was available for the MBL and SFTPD measurements, and (3) if allergic-bronchopulmonary aspergillosis and acute pulmonary exacerbation or infection within 4 weeks preceding the time of blood sampling were ruled out. Forced expiratory volume in 1 s expressed as percentages of predicted values (FEV1%), age at diagnosis of CF, gender, CFTR genotype as well as age at detection of first positive result for PA culture and age at transition to chronic PA colonization were collected retrospectively from the medical records. CF patients without detection of PA in cultures were assigned as negative (PA-) and those with any detection of PA strains as positive (PA+). According to the “Leeds criteria,” chronic Pseudomonas infection (PAC) was diagnosed if >50% of cultures taken over a minimum 12 months period were positive for PA and intermittent Pseudomonas infection (PAI) was diagnosed if ≤50% of cultures were positive (23).

### Blood Sampling and Processing

Venous whole blood samples were collected for research purposes in addition to those taken for routine clinical care of the patient. After complete clotting at room temperature the samples were centrifuged within 1 h (1,500 × *g* at 4°C for 15 min). The serum was carefully separated from the blood clot and aliquoted into plain polypropylene tubes. Serum aliquots and the remaining blood clot were stored at −80°C.

### MBL and SFTPD Serum Concentrations

Serum concentrations of SFTPD and MBL were measured by commercially available enzyme-linked-immunosorbent-assay (ELISA) according to the manufacturer's protocol (MBL Human-ELISA kit HK323 and SFTPD Human-ELISA kit HK335, Hycult Biotech, Uden, Netherlands). All assays were performed in duplicate and values were accepted with a coefficient of variation <5%.

### Genotyping Methods

Genomic DNA was extracted from frozen clotted blood samples with commercial DNA isolation Kit (QIAamp DNA Mini Kit, Qiagen, Hilden, Germany) for PCR reactions. The allelic discrimination was performed on an Applied Biosystems 7500 Real Time PCR System (Foster City, CA) and all reactions were carried out according to the manufacturer's instructions. The TaqMan 5‘nuclease method (Assays-on-Demand, Applied Biosystems, Darmstadt, Germany) was used for analyzing reported functional polymorphisms. For the MBL2 coding gene we genotyped functional relevant SNPs located in the promoter region at position−221G>C (rs7096206, allele X/Y) and in exon 1: 219C>T transition at codon 52 (rs5030737, Arg52Cys, allele D), 226G>A transition at codon 54 (rs1800450, Gly54Asp, allele B), and 235G>A transition at codon 57 (rs1800451, Gly57Glu, allele C). These three structural variants in exon 1 of the collagen-like domain are believed to affect the oligomer formation of MBL, resulting in impaired protein function and stability (24). The variant alleles B, C, and D are collectively referred to as O, whereas the wild-type allele is termed as A. The serum concentration of MBL is further influenced by the SNP−221X/Y in the promoter region (25). On the basis of the previously described strong linkage disequilibrium between the structural and promoter polymorphisms we combined the MBL2 gene variants into the following well-characterized haplotypes, which modulate serum concentrations of high-order MBL oligomers. The haplotypes YAYA, YAXA are associated with high expression, whereas YAYO, YOYO, XAYO, YOXO, and XAXA are associated with low expression of the MBL protein (24). For SFTPD we tested three SNPs located in coding regions and with minor allele frequencies >5% in Caucasian populations: Met11Thr (rs721917, 135T>C), Ala160Thr (rs2243639, 581G>A), and Ser270Thr (rs3088308, 911T>A) (26, 27).

### Statistical Analysis

The statistical analysis was performed using IBM SPSS Statistics version 20 software (Armonk, New York, United States). Statistical significance was defined at the minimal two-tailed *p*-value of 0.05. Descriptive statistics for quantitative and qualitative values are given by mean with standard deviation and absolute numbers with frequencies. The non-parametric Mann-Whitney *U*-test and Kruskal-Wallis *H*-test were used to assess differences in continuous variables between groups. For *post-hoc* pairwise testing we used Dunn's non-parametric method. The Spearman's rank test was used to determine the correlations between serum collectin levels and FEV1%. The significance of proportional differences in haplotypes and genotype were determined by using the Fisher's exact test as expected counts were small. Age at first infection with PA, length of period between first PA infection and onset of chronic PA colonization as well as age at transition to PAC were compared between groups by Kaplan-Meier survival curve analysis with log-rank test. We used the SHEsis software (http://analysis.bio-x.cn) to evaluate the pairwise linkage disequilibrium coefficients *D*‘and *r*^2^ between the studied alleles in the SFTPD locus. SFTPD haplotype estimation was performed by the expectation maximization method using HPlus 2.5 software (http://qge.fhcrc.org/hplus/). Multivariate cox regression analysis was done using HPlus software. Obtained hazard ratios (HR) are given with 95% confidence intervals (95%CI) and two-sided *P*.

## Results

### Study Population

In total, 35 out of 76 caucasian patients homozygous for ΔF508del CFTR mutation could be included in the study (20 (57.1%) male and 15 (42.9%) female). The mean age at last follow-up was 18.8 ± 5.0 years and did not differ between PA groups (*p* < 0.48). Table 1 lists demographics and key clinical characteristics of participants according to the PA infection status.

**Table 1:** Clinical features of the study population.

	**PAC (*n* = 15)**	**PAI (*n* = 12)**	**PA- (*n* = 8)**	***p*-value**^**a**^	***p*-value**^**b**^
Male gender, *n* (%)	8 (53.3%)	7 (58.3%)	5 (62.5%)	0.91	n.s.
FEV1%, percentage predicted	67.8 ± 18.5	70.7 ± 25.4	88.1 ± 9.6	0.04^*^	0.07
Age at diagnoses of CF (years)	1.0 ± 1.3	1.5 ± 2.0	1.6 ± 2.3	0.84	n.s.
Age at blood sampling (years)	11.5 ± 4.2	11.2 ± 6.5	12.5 ± 4.9	0.81	n.s.
Age at last follow-up (years)	19.7 ± 5.3	17.4 ± 5.0	19.0 ± 4.4	0.45	n.s.
Age at first documented culture (years)	9.3 ± 4.9	8.5 ± 6.5	11.9 ± 5.2	0.24	n.s.
Age at first PA detection (years)	10.2 ± 5.4	12.7 ± 6.1	.	0.22	.

*Values are given as number with percentages or mean with standard deviation. FEV1%, forced expiratory volume in 1 s*.

a PA-, PAI and PAC groups compared by Fisher's exact test or by Kruskal-Wallis-H test with post-hoc Dunn's pairwise analysis (^*^PAC vs. PA-).

b *PA- vs. PAC+PAI (=PA+) using Fisher's exact test or Mann-Whitney-U test*.

* *Significant at p < 0.05*.

Overall, 27 (77.1%) patients were infected with PA during the study period and in 15 (55.6%) of whom the infection was chronic (PAC). Neither the rate of PA infection (75 vs. 80%, *p* < 0.70) and of PAC (40.1 vs. 46.7%, *p* < 0.69) nor the median age at first PA culture (14.0 vs. 11.7 years, *p* < 0.50) and age at onset of PAC (25.9 vs. 18.6 years, *p* < 0.51) showed statistically significant differences between male and female patients. PA airway infection negatively influenced the pulmonary function recorded as FEV1% at last follow-up. The FEV1% values of patients with PAC was lowest and differed significantly only in comparison to never PA infected patients (*p* < 0.04). All studied polymorphisms in the MBL2 and SFTPD coding genes did not deviate from the distributions predicted by the Hardy-Weinberg equation as shown in Table 2.

**Table 2:** Distribution of genotypes in the study cohort.

**Gene**	**SNP**	**Genotypes**	**Frequencies**	**HWE *p*-value**
MBL2	−221G>C	CC – CG – GG	2 (5.7%) – 11 (31.4%) – 22 (62.9%)	0.69
	219C>T	CC – CT – TT	26 (74.3%) – 9 (25.7%) – 0 (0%)	0.38
	226G>A	AA – GA – GG	0 (0%) – 8 (22.9%) – 27 (77.1%)	0.45
	235G>A	AA – GA – GG	0 (0%) – 2 (5.7%) – 33 (94.3%)	0.86
SFTPD	135T>C	CC – TC – TT	2 (5.7%) – 16 (45.7%) – 17 (48.6%)	0.48
	581A>G	AA – AG – GG	7 (20.0%) – 18 (51.4%) – 10 (28.6%)	0.83
	911T>A	AA – TA – TT	0 (0%) – 3 (8.6%) – 32 (91.4%)	0.79

*HWE, Hardy-Weinberg equlilibrium. Data are n (%)*.

### Mannose-Binding Lectin

**Figure 1:**
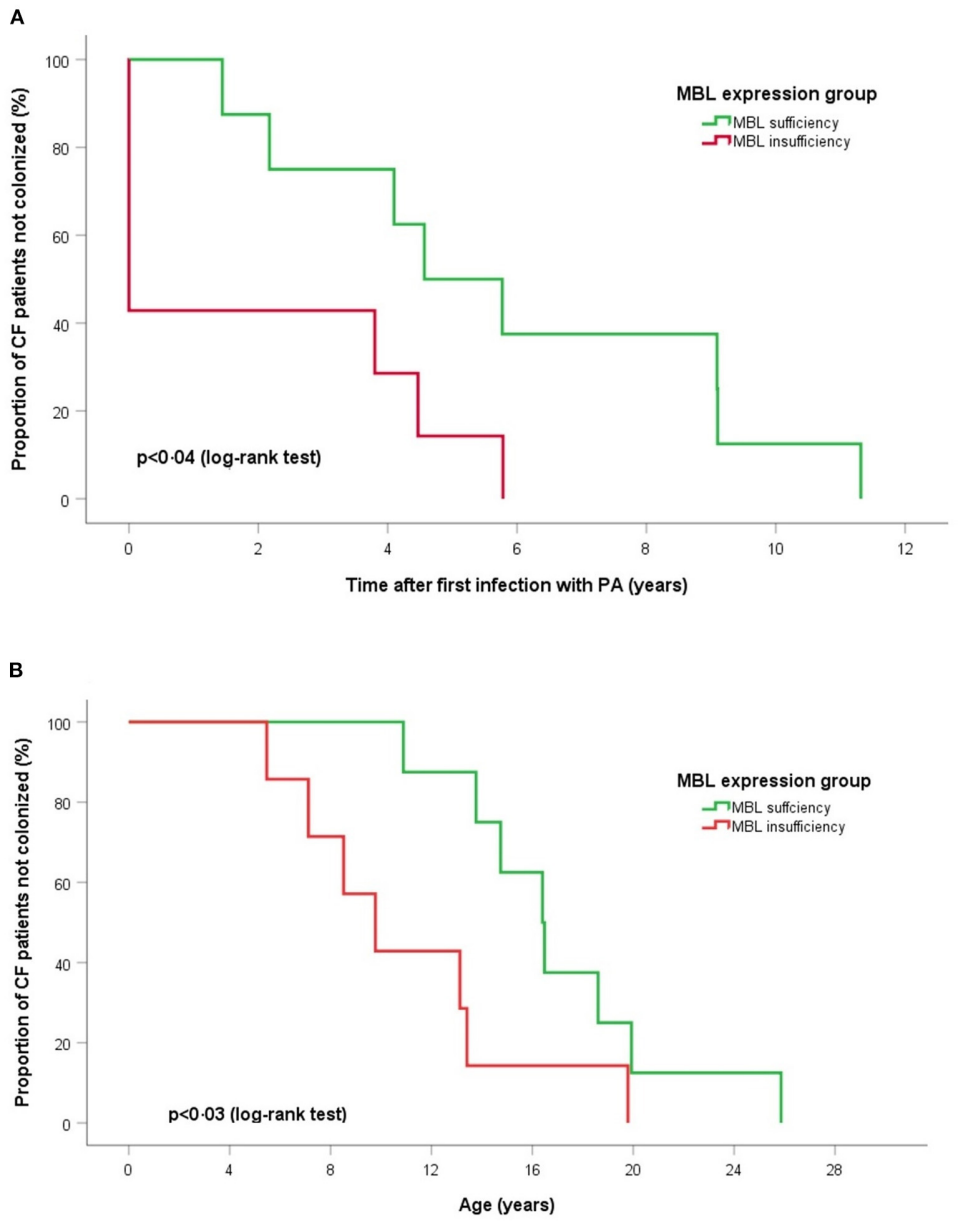
Log-rank tests and Kaplan-Meier curves of time for chronic PA infection according to MBL expression groups. **(A)** Time after first PA acquisition and **(B)** age of onset chronic PA colonization. Log-rank tests show for MBL sufficient producers a significant longer duration between first acquisition and start of PA colonization (*p* < 0.04) and an older age at onset of chronic PA infection (*p* < 0.03).

Extended MBL2 genotypes were merged according to the MBL expression into MBL sufficient (YAYA, YAXA) and MBL insufficient groups (YAYO, YOYO, XAYO, YOXO, and XAXA): 16 (45.7%) patients were considered MBL sufficient and 19 (54.3%) MBL insufficient (Table 3). There were no differences between sufficient and insufficient MBL groups according to the mean age at last follow-up (20.2 ± 5.1 vs. 17.5 ± 4.7 years, *p* < 0.11), number of individuals positive for PA (68.8 vs. 84.2%, *p* < 0.42) and PAC (50.0 vs. 36.8%, *p* < 0.51). Kaplan-Meier curves and log-rank test revealed a similar median age at first (*p* < 0.52) and at onset of chronic PA infection (*p* < 0.92) between both MBL expression groups. The subgroup analysis of patients with chronic PA colonization (*n* = 15) has found that CF individuals with MBL insufficiency have both a shorter interval between first PA infection and onset of PAC (0.01 vs. 4.6 years, *p* < 0.04) as well as a lower median age at transition to chronic PA colonization (9.8 vs. 16.4 years, *p* < 0.03) compared to MBL sufficient patients (Figure 1). Irrespective of the PA infection status, the FEV1% values did not vary significantly between MBL sufficient and MBL insufficient producers (72.9 ± 24.0 vs. 73.5 ± 25.2%, *p* < 0.94). As expected, the serum levels of MBL were highly related to MBL expression groups (269 ± 126 vs. 74 ± 98 ng/ml, *p* < 0.001). Overall, patients age, gender, and PA status did not affect MBL serum concentrations (data not shown).

**Table 3:** Distribution of MBL2 haplotypes and expression groups in CF patients.

	**All patients (*n* = 35)**	**PAC (*n* = 15)**	**PAI (*n* = 12)**	**PA- (*n* = 8)**
**MBL sufficiency**	**16 (45.7%)**	**8 (53.3%)**	**3 (25.0%)**	**5 (62.5%)**
- YAYA	9 (25.7%)	3 (20.0%)	2 (16.7%)	4 (50.0%)
- YAXA	7 (20.0%)	5 (33.3%)	1 (8.3%)	1 (12.5%)
**MBL insufficiency**	**19 (54.3%)**	**7 (46.7%)**	**9 (75.0%)**	**3 (37.5%)**
- YAYO	11 (31.4%)	3 (20.0%)	6 (50.0%)	2 (25.0%)
- XAYO	4 (11.4%)	1 (6.7%)	2 (16.7%)	1 (12.5%)
- XAXA	2 (5.7%)	2 (13.3%)	0 (0%)	0 (0%)
- YOYO	2 (5.7%)	1 (6.7%)	1 (8.3%)	0 (0%)
- YOXO	0 (0%)	0 (0%)	0 (0%)	0 (0%)

*Data are n (%). No significant differences were found in distribution of haplotypes among the study groups (all p > 0.05 tested by Fisher's exact test)*.

### Surfactant Protein-D

We found no association between the genotype distribution of SNPs in SFTPD and the PA infection status (Table 4). None of studied SFTPD polymorphisms showed an effect on age at first and at onset of chronic PA infection (Kaplan-Meier curves with log-rank test). Mean serum SFTPD level was 863 ± 389 ng/ml in our cohort of CF patients. Age, gender, PA infection status, and SFTPD polymorphisms did not influenced SFTPD serum concentrations (data not shown). SFTPD serum level correlated negatively with FEV1% values (Spearman *r* = −0.41, *p* < 0.03). All studied SNPs within the SFTPD coding gene were in linkage disequilibrium (pairwise *D'*and *r*^2^ coefficients): *D'* = 0.84/*r*^2^ = 0.22 for Met11Thr and Ala160Thr; *D'* = 0.99/*r*^2^ = 0.11 for Met11Thr and Ser270Thr; *D'* = 1.0/*r*^2^ = 0.05 for Ala270Thr and Ser270Thr. From the three polymorphisms three major haplotypes were reconstructed, accounting for about 94% of all haplotypes observed in our study cohort (Table 5). We investigated the relationship between age at onset of chronic PA infection and SFTPD haplotypes. Taking the common T-A-T haplotype as reference, the cox proportional hazards regression analysis confirmed an association of C-G-T haplotype with an earlier age at onset for PAC [HR adjusted for gender 3.0 (95%CI: 1.1–8.6), *p* < 0.04].

**Table 4:** Distribution of genotypes of the SNPs in the SFTPD coding gene in CF patients.

	**Genotype**	**PA-**	**PAI**	**PAC**	*p*-value^*^
Met11Thr	TT	4 (50%)	7 (58.3%)	6 (40%)	0.52
	TC	4 (50%)	5 (41.7%)	7 (46.7%)	
	CC	0 (0%)	0 (0%)	2 (13.3%)	
Thr160Ala	AA	3 (37.5%)	2 (16.7%)	2 (13.3%)	0.63
	AG	4 (50%)	6 (50%)	8 (50.3%)	
	GG	1 (12.5%)	4 (33.3%)	5 (33.3%)	
Ser270Thr	TT	8 (100%)	11 (91.7%)	13 (86.7%)	0.55
	TA	0 (0%)	1 (8.3%)	2 (13.3%)	
	AA	0 (0%)	0 (0%)	0 (0%)	

*Data are n (%)*.

* *Compared by Fisher's exact test*.

**Table 5:** Estimated major SFTPD haplotypes and cox proportional hazard regression analysis for age at onset of PAC (adjusted for gender) using HPLus 2.5 software.

**Haplotype**	**Frequency (SE)**	**Coefficient (SE)**	**HR**	**95%CI**	***Z*-score**	***p*-value**
TAT	0.44 (0.52)		1.00			
TGT	0.29 (0.53)	0.95 (0.61)	2.59	0.79–8.55	1.56	0.12
CGT	0.21 (1.05)	1.10 (0.54)	3.01	1.05–8.62	2.05	0.04

*HR, hazard ratio; SE, standard error; 95%CI, confidence interval of HR*.

## Discussion

A notable number of patients with CF experience a more difficult clinical course associated with an increased hospitalization rate and an early lung deterioration. The etiology behind the frequency of pulmonary exacerbations and its associated poorer prognosis is focus of different research approaches. An immense effort was invested in trying to understand causes of early deterioration and susceptibility to infection of the lungs. According to clinical studies, the leading determinant of clinical outcome is microbiological colonization (28, 29). Surfactant proteins A (SFTPA) and D (SFTPD) have been implicated in pulmonary innate immunity. The proteins are host defense lectins, belonging to the collectin family which also includes mannan-binding lectin (MBL) (30).

In our study, we analyzed whether there is an association between SNPs in the SFTPD and MBL coding genes and the age at PA infection. In the present paper we examined the association between three functional polymorphisms within the SFTPD gene and serum SFTPD concentrations. Furthermore, we investigated for the first time the influence of SFTPD haplotypes on susceptibility of PA infection in CF patients.To our best knowledge this is the first study to describe and analyze SFTPD as a genetic modifier in onset of chronic PA infection in CF patients.

In accord with published data we were able to describe an association between MBL insufficiency and onset of chronic PA colonization in a subgroup of CF patients (12). There are variations in phenotype and severity of CF even if the cause is the same CTFR mutation. MBL has been proposed as one of these factors and has been well-investigated during the last decades. Despite MBL deficiency not being the cause of cystic fibrosis, several studies have shown the impact of MBL expression and polymorphisms on the course of CF disease (13). Similar findings were found in patients with non-CF bronchiectasis and in patients with primary ciliary diyskenesia (31–33). Our data shows that serum levels of MBL were highly related to MBL haplotypes with MBL serum concentrations being unaffected by patients age, gender, and PA status. These results are in agreement with published research and demonstrates that patient cohort does not differ from other research studies (34). Some studies showed that FEV1% values can decline significantly in MBL insufficient producers patients, however this is not supported by our data which might be due to the relatively small cohort in our study (35). Moreover, the utility of FEV1% in assessing CF patients is limited since the reproducibility is reduced in patients with a severely restricted lung function (36). We found that those CF patients with MBL insufficiency have both a shorter interval between first PA infection and onset of PAC as well as a lower median age at transition to PAC colonization compared to MBL sufficient patients. The results outline and support the meaning of mannose-binding lectin as a disease modifier in CF.

SFTPD, as member of the collectin family, such as SFTPA and MBL, has important anti-microbial and anti-inflammatory functions (37). It plays a crucial role both in the local clearance of respiratory pathogens and in the modulation of pulmonary inflammation (37). Serum SFTPD has been suggested as a systemic biomarker for prognosis and outcome of pulmonary disoders (19). In patients with CF, the serum SFTPD was found to be increased in parallel with infection and with reduced lung function (18, 21). The serum SFTPD levels in our patient cohort were slightly reduced compared to reported values in CF patients in the study of Oelsen et al. but higher than published levels from healthy pediatric controls (10). We found no correlation between SFTPD serum concentrations and status of PA infection within our patient cohort. Our data confirm the known inverse association between serum SFTPD and limited lung function measured as FEV1%. Reduced concentrations of SFTPD in the bronchoalveolar lavage fluid (BALF) are associated with impaired lung function and increased SFTPD levels in serum correlated with inflammation and lung damage. Low levels of SFTPD have been demonstrated in BALF of CF patients compared with healthy controls (19). This might result from proteolytic degradation and increased leakage of endogenous SFTPD from the lung into the circulatory system due to an impaired alveolar-capillary permeability (38). The damage and down regulation of SFTPD in the CF lung seem to be inversely related to the intensity of local inflammatory activity and positive PA infection status (20). The functional capabilities of SFTPD are strongly linked to their degree of multimerization, which have crucial implications for patients with chronic lung disorders such as CF (15, 39, 40). Only high multimeric forms of pulmonary SFTPD exhibit a robust mannan binding activity (20, 41). There is growing evidence that structural SFTPD polymorphisms influence constitutional serum level, lectin activity and the state of SFTPD oligomerization. Both in health and lung disease, carriers of the amino acid methionine at codon 11 within the SFTPD gene show highest serum concentrations of SFTPD, but more importantly they exhibit a multimeric organization of SFTPD with an unrestricted lectin binding activity compared to counterparts carrying threonine at position 11 (42, 43). The agglutination of PA was shown to correlate with the concentration and multimeric organization of SFTPD in BALF (44). Genetic associations between polymorphisms of SFTPD and various pulmonary diseases have been reported (22, 37). Polymorphisms in the SFTPD gene are shown to be associated with increased risk of bacterial and viral infections (45, 46). To date, the few studies investigating surfactant protein genes in CF disease could not demonstrate a substantial link with SFTPD polymorphisms (18, 37). Olesen et al. could neither confirm the reported association between the SFTPD Met11Thr variation and serum SFTPD for the complete CF patient population, nor found any evidence for the single-marker SFTPD variant and lung function. Like previous studies our investigations also showed, that none of the three single SNPs in the SFTPD encoding gene is linked to serum SFTPD levels, lung function and PA infection in CF patients. The genotype distribution of each SNP is neither associated with status nor with age at first detection or at onset of chronic PA infection. However, genetic association studies are complicated by the fact of existing strong linkage disequilibrium between alleles, which form haplotypes across the gene with relevant multilocus effects not seen for single variants. From the three studied SNPs in the SFTPD encoding gene three major haplotypes were reconstructed, accounting for about 94% of all haplotypes observed in our study cohort. Our data about the three-point haplotypes consisting of the Met11Thr, Ala160Thr, and Ser270Thr polymorphisms are in good accordance with previously published results from a Caucasian control population (47). We investigated the relationship between age at onset of chronic PA infection and SFTPD haplotypes. Taking the common T-A-T (=11Met-160Thr-270Ser) haplotype as reference, regression analysis demonstrated a significant association of the C-G-T (=11Thr-160Ala-270Ser) haplotype with an earlier age at onset for PAC. In the present study, we found that in multilocus analysis of SFTPD, three SNP haplotype T-A-T was significantly associated with reduced risk of chronic PA infection. Our results suggest the involvement of T-A-T haplotype in delaying PAC infection.This might be mediated by the presence of one allele with methionine at codon 11 which influences the oligomerization of SFTPD and thereby its anti-microbial function against PA. The relatively low number of enrolled patients could be considered a major limitation of our study. We did not simultaneously obtain BALF, as we tried to be as less invasive as possible in patients with progressive disease. Importantly, our subjects were accurately selected regarding Caucasian ethnicity and homozygosity for the ΔF508del mutation in the CFTR gene in order to minimize their clinical heterogeneity. Future studies should determine the high and low forms of serum SFTPD as well as the analysis of SFTPD levels in BALF of CF patients.

In addition, several other polymorphisims of MBL2 and SFTPD gene, despite those being analyzed in our study, have been proved and described as functionally important in other diseases and recurrrent infections (48–51). Future reseach should include all of these SNPs to provide a comprehensive locus wide association of genes.

## Conclusion

Our study supports the fact that CF patients with MBL insufficiency have both a shorter interval between first PA infection and onset of PAC as well as a lower median age at transition to PAC colonization compared to MBL sufficient patients. Our results emphasize the importance of mannose-binding lectin as a secondary disease modifier in CF. Our study is the first one to be able to demonstrate that SFTPD haplotypes could modulate the susceptibility for PA infection in CF patients showing its importance as an additional disease modifier and a potential prognostic biomarker in patients with CF.

## Data Availability Statement

The raw data supporting the conclusions of this article will be made available by the authors, without undue reservation.

## Ethics Statement

All protocols used in this study were approved by the local ethics committee (file no. 220/05).

## Author Contributions

NN-T, ET, KF, and MZ designed the research and interpreted the results. NN-T, ET, and KF coordinated data collection, performed lab analyses, and analyzed the data. NN-T wrote the manuscript. All authors reviewed the manuscript.

## Conflict of Interest

The authors declare that the research was conducted in the absence of any commercial or financial relationships that could be construed as a potential conflict of interest.

## Abbreviations

CF: Cystic Fibrosis
CTFR: Cystic fibrosis transmembrane conductance regulator
PA: *Pseudomonas aeruginosa*
SFTPD: Surfactant Protein-D
SFTPA: Surfactant Protein-A
BALF: Bronchoalveolar lavage fluid
MBL: Mannose-binding lectin
FEV1%: Forced expiratory volume in 1 second percentage predicted.
